# Fracture-based grasping: dynamic impact enables predictable robotic anchoring to freshwater ice

**DOI:** 10.1038/s44182-026-00085-0

**Published:** 2026-03-27

**Authors:** Andrew Galassi, Ashitey Trebi-Ollennu, Panayiotis Papadopoulos, Hannah S. Stuart

**Affiliations:** 1 Department of Mechanical Engineering, University of California, Berkeley, CA USA; 2 Jet Propulsion Laboratory, California Institute of Technology, Pasadena, CA USA

**Keywords:** Engineering, Materials science

## Abstract

Gripping to smooth and wavy substrates, such as naturally occurring ice, presents a challenge for climbing robots in the field. Existing ice anchoring solutions require either substantial initial surface compression force (drilling; at least 50 Newtons) or require large energy expenditure (thermal picks; almost 1000 Joules). We present an anchoring mechanism capable of attaching to ice with lower initial surface compression force and lower energy consumption compared to drill-based or melt-based methods. The system leverages surface fracture caused by dynamic impacts with axes – inspired by mountaineers – to create indentations for grasping. A model describes the indentation depth, recoil energy, and surface compression force required for anchoring success, each as a function of impact energy. An integrated dual-ax gripper system successfully generates usable indents with as low as 8.3 Newtons of initial surface compression force and 8 Joules of combined mechanical potential energy on -14^∘^ C freshwater ice – a result consistent with first-principle model predictions. The gripper then successfully holds its own weight on steep glacier slopes in the field. These results indicate fracture-based grasping approaches are promising for climbing systems on ice. This concept can also apply to other surfaces such as wood, rock, and packed soil.

## Introduction

The icy worlds of Enceladus and Europa represent two compelling science targets in the outer solar system. With subsurface liquid water oceans^1,2^ and active thermal energy sources^3–5^, these small moons represent some of the best places in the solar system to search for extant extraterrestrial life. Enceladus is particularly compelling for its active plumes^6^, depositing material onto the surface, and potentially providing a natural access point to the subsurface ocean. Recent findings reinforce the potential for habitability in the ocean of Enceladus: evidence suggests that the ocean is warm and briny, contains simple hydrocarbons and methane^4,7^ and contains nearly all the basic elements required for life, including carbon, hydrogen, nitrogen, oxygen, and phosphorus^7–9^. Enceladus poses unique challenges for robotic explorers, however. The gravitational acceleration at the surface of Enceladus is low (0.113 m/s^2^^10^, or 1% Earth gravity). For robotic climbers to descend the active vents and sample the ocean directly, a vehicle must be able to withstand the force of the plume ejecta, which may far exceed the weight of the vehicle itself^11^. As a result, a robotic climbing vehicle for Enceladus would likely need to (i) form a strong attachment to the icy terrain and (ii) do so with minimal initial surface compression force against the surface. Furthermore, the icy shell could be as thick as tens of kilometers near the vent openings^6^. Thus, such a vehicle would need to (iii) be able to repeatedly anchor with minimal energy expenditure for each anchor formed, as it grasps and ungrasps the surface with each step.

Two notable prior methods for robotic anchoring to ice include ice screws and thermal picks. Ice screws – such as for commercial climbing tools used by human ice climbers – thread into the ice, forming a strong attachment to the surface. Ice screw end effectors have been deployed in Earth-analogue field tests, demonstrating strong anchoring for vertical robot mobility^12^. However, initial engagement of these anchors were shown to necessitate as much as 200 N of weight-on-bit force. Achieving this force with vehicle weight alone on Enceladus would require a 2000 kg vehicle, roughly twice the mass of the Perseverance rover^13^. Thermal anchoring consists of a single heated pick or multiple heated prongs sublimating the surface to form an anchor. These mechanisms can anchor with low insertion force, or less than 10 Newtons, to engage and have been demonstrated on Earth, functioning in both dry ice and water ice surface simulants^14,15^. While effective for anchoring with low initial compression force, strong thermal anchoring is energy intensive, on the order of 8 kJ per insertion, modeled in a Europa environment^15^, or about 1 kJ on Earth. The repeated engagement of thermal anchors for robotic mobility could represent prohibitively large power consumption for severely energy-constrained vehicles in the outer solar system.

Utilizing dynamic impact and subsequent grasping to anchor to the icy substrate could result in a relatively low initial compression force to anchor, while reducing energy consumption compared to the state of the art, and form a strong anchor to ice. This method of attachment is common to human mountaineers using ice axes, but not yet common in their robotic counterparts. Table 1 compares anchoring through thermal picks and ice screws with the results of the present work using ice axes; we find that ice ax impact anchoring achieves more favorable values for both estimated minimum energy per anchor and compression required.

**Table 1:** Comparison of ice anchoring mechanisms

Anchoring Mechanisms	Visual Representations	Specific Energy (MJ/m^3^)	Volume Removed (m^3^)	Estimated Energy per Anchor (J)	Initial compression Force Needed (N)
Ice screw^12^		0.46	2 × 10^−5^	9.2	50 to 200
Thermal pick^14^		300	3 × 10^−6^	930	6.5 to 40
Ice ax impact		0.46	9 × 10^−7^	0.40	8 to 16

Table comparing the relevant ice properties, volume removed, estimated minimum energy required per anchor, and compression required for thermal anchoring, ice screw drilling, and impact anchoring. The specific energy for ice under rotary drilling and impact anchoring is assumed to be the estimated toughness of Enceladus ice^16^. Thermal pick estimates uses the latent heat of fusion of ice.

This hypothesis is informed by the mechanical properties of freshwater ice. The latent heat of fusion of water is extremely high relative to its energetic toughness when solid; while the latent heat of fusion of water is around 300 MJ/m^3^, its energetic toughness is less than 1 MJ/m^3^ in the case of simulated Encleadus plume deposits^16^. Even in the case of Encleadus ice having some porosity, this orders-of-magnitude difference indicates the energetic benefit of fracture-based methods over melt-based methods. Among fracture based methods, drilling and impacting are divided by their relative strain rates. Dynamic impact has a relatively higher strain rate than rotary drilling. While drilling is related to material strength, impact dynamics are more related to the fracture toughness of ice. The tensile strength and fracture toughness of freshwater ice are both relatively low, on the order of 1 MPa and 0.1 MPa/m^1/2^, respectively^17^. While both methods are relatively energy efficient compared to melting, the rate-dependence of the fracture toughness of ice presents an opportunity because it decreases significantly with increasing stress intensity factor rate^18^. Therefore, for a given expended energy, dynamic impact with a high strain rate should fracture a larger volume of material than drilling with a lower strain rate. As a result, impact with stored energy could serve as an energy efficient method of ice fracture to enable subsequent anchoring.

**Fig. 1:**
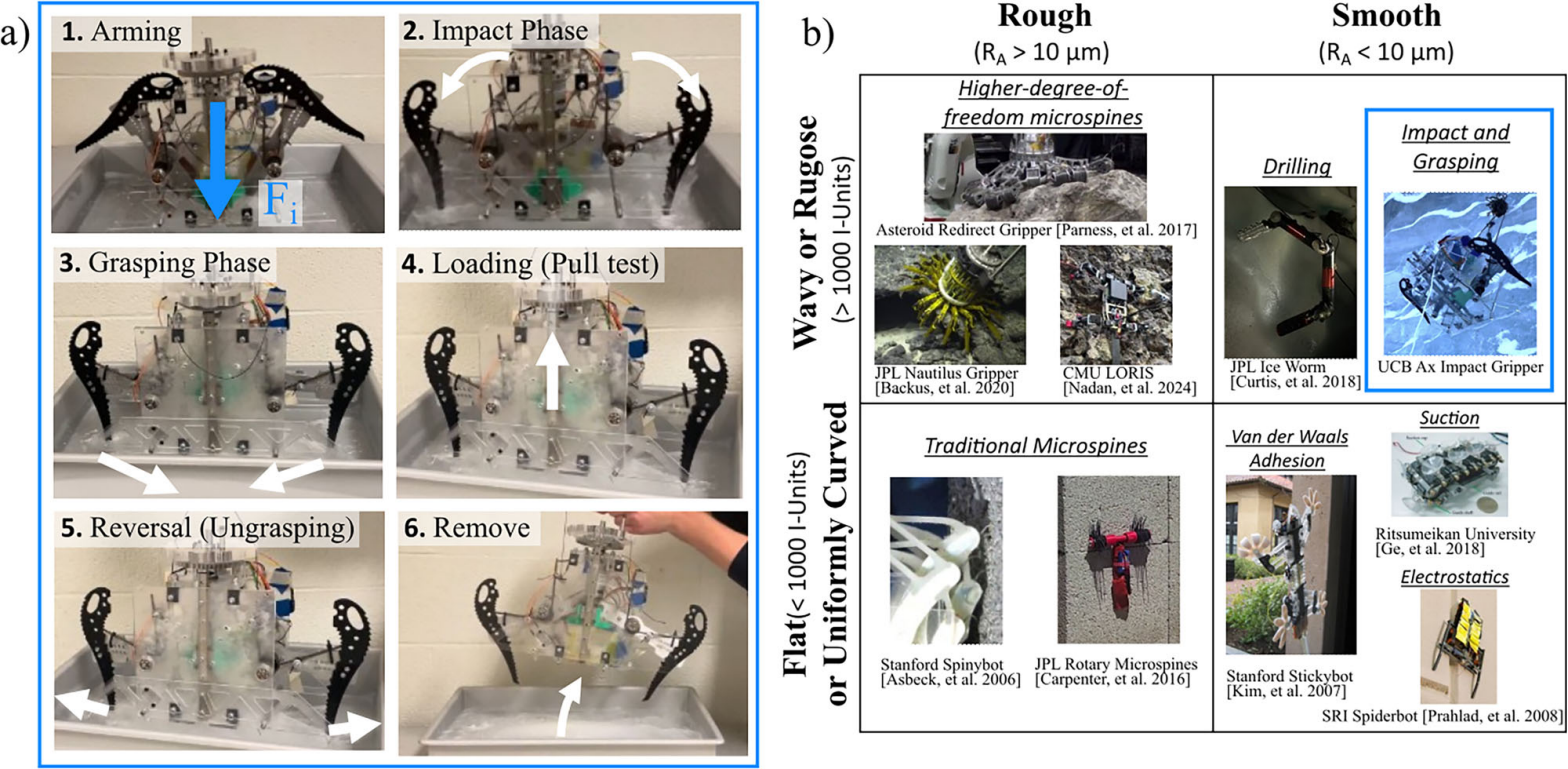
An overview of our impact and grasping-based anchoring approach and a comparison of anchoring technologies use in robotic mobility versus surface properties. **a** Impact and grasp operational stages are demonstrated, with initial compression force shown as *F*_*i*_. **b** Grasped-surface properties contextualize our anchoring approach among existing anchoring technologies used previously in mobile robots. A flat substrate, as opposed to one that is wavy or rugose, is defined as having an I-Unit value of less than 1000, whereas wavy or rugose is defined as an I-Unit value of greater than 1000. I-Unit value is a standard measure of deviation from flatness and is proportional to the square of the ratio of periodic wave height to length. A rough surface is defined as having an *R*_*A*_ -- arithmetic average of absolute height deviations from a mean surface line -- value of greater than 10 *μ*m (order of magnitude), whereas a smooth surface would have an *R*_*A*_ value of less than 10 *μ*m. We use the two-dimensional line scan roughness parameter *R*_*A*_, as opposed to areal surface parameters, as prior spine literature commonly cites it.

Figure 1a shows the concept of operation for impact anchoring using two ice axes. This method requires not only an initial impact phase, but also a grasping phase to engage the resulting indentations formed to create a strong anchor to the surface. We refer to this overall anchoring process as anchoring via impact and grasping. First, the mechanism stores energy in two torsion spring-loaded arms, with sharpened ice axes at the ends. The energy is then released, and imparted into the surface during the impact phase. The grasping phase then introduces an internal grasp force between the indenting ax tips; this is ultimately what causes the strong attachment. During experiments, a load test is then conducted to quantify the anchor strength normal to the surface, when the gripper separates from the surface. However, the figure illustrates that the anchor can easily reverse its anchor by releasing the grasp force, allowing for removal of the anchor and resetting of the spring loading mechanism. All mechanism functions for this reversible and repeatable anchoring gripper are performed by a single linear actuator, housed within the mechanism. To our knowledge, the results represent the first instance of impact fracture of a substrate enabling a robot to grip to a ice surface. Video demonstrations of operation can be found in Supplementary Movie 1.

Figure 1b contextualizes our work among varying substrates for anchoring and technologies for robotic anchoring for mobility that exist. Substantial work enables mobility on rough surfaces. Microspine technology is utilized on substrates with substantial surface asperities. They have been shown functioning successfully on concrete and brick^19^, vehicular basalt^20,21^, windswept sandstone^22^, and other materials^23–25^. Despite their versatility, they depend on preexisting surface features to form their anchor, and have stochastic anchoring performance^26^. While early iterations of microspine technology enabled anchoring to surfaces that were flat on a macro scale^27^, it is possible to design higher-degree-of-freedom mechanisms to enable anchoring to irregular rough surfaces^28,29^. However, these higher-degree-of-freedom mechanisms still depend on either preexisting surface asperities, or force or form closure with an object smaller than the mechanism linkages in order to form an attachment^30^. For smooth surfaces with low surface roughness, gecko-inspired adhesives are commonly used. It is possible for a surface to be too smooth for optimal attachment with gecko-inspired adhesives, but the experimentally-determined optimal range of surface roughness lies orders of magnitude below the surface roughness required for microspine anchoring^31^. Due to their morphology, gecko-inspired adhesives are most effective anchoring on surfaces that are either flat^32^ or uniformly curved^33^. While it can effectively supplement other gripping strategies for irregular objects smaller than the hand^34^, wavy surfaces present a challenge. Among the category of climbing robots limited to surfaces that are both smooth and flat or uniformly curved, there are also suction climbers^35^, and electrostatic adhesion-based climbers^36^. These approaches would face similar challenges as gecko adhesives, in that they rely on contact area and need engineering suspensions to distribute their contact across wavy surfaces.

Reversible anchoring to the category of substrates which are both smooth (low surface roughness) and wavy (low flatness) has not been demonstrated in mobile robots as extensively. We propose an approach to anchoring to these substrates by creating asperities in a smooth surface, instead of adapting area-sensitive technologies to wavy surfaces. Our work achieves this by applying the approach of impact fracture and grasping to the specific substrate of freshwater ice.

## Results

An energy-based model accurately predicts indentation depth and approximates worst-case recoil for a single ax impacting freshwater ice. We then show that this model provides a first-principles estimate of the surface preload compression needed – 16 Newtons – for 99.8% anchoring success with the complete dual-ax gripper. Upon grasping, we find that two anchor strength failure modes emerge related to the grasp force applied: slip out of the indents or fracture of the indent ledges. The maximum anchor strength normal to the surface is empirically observed as 75 Newtons, or over 2 times the weight of the gripper, with an internal grasp force of 175 Newtons. All of these laboratory trials were conducted on -14 ^∘^ C freshwater ice, selected as an analog to a glacier environment, which was the subject of the field test for this work. Field trials demonstrate the anchoring capability of the gripper on natural glacier ice, consistent with predicted results, as well as its versatility to also attach to tree trunks, rocks, and compacted dirt.

### Impact phase of anchoring

**Fig. 2:**
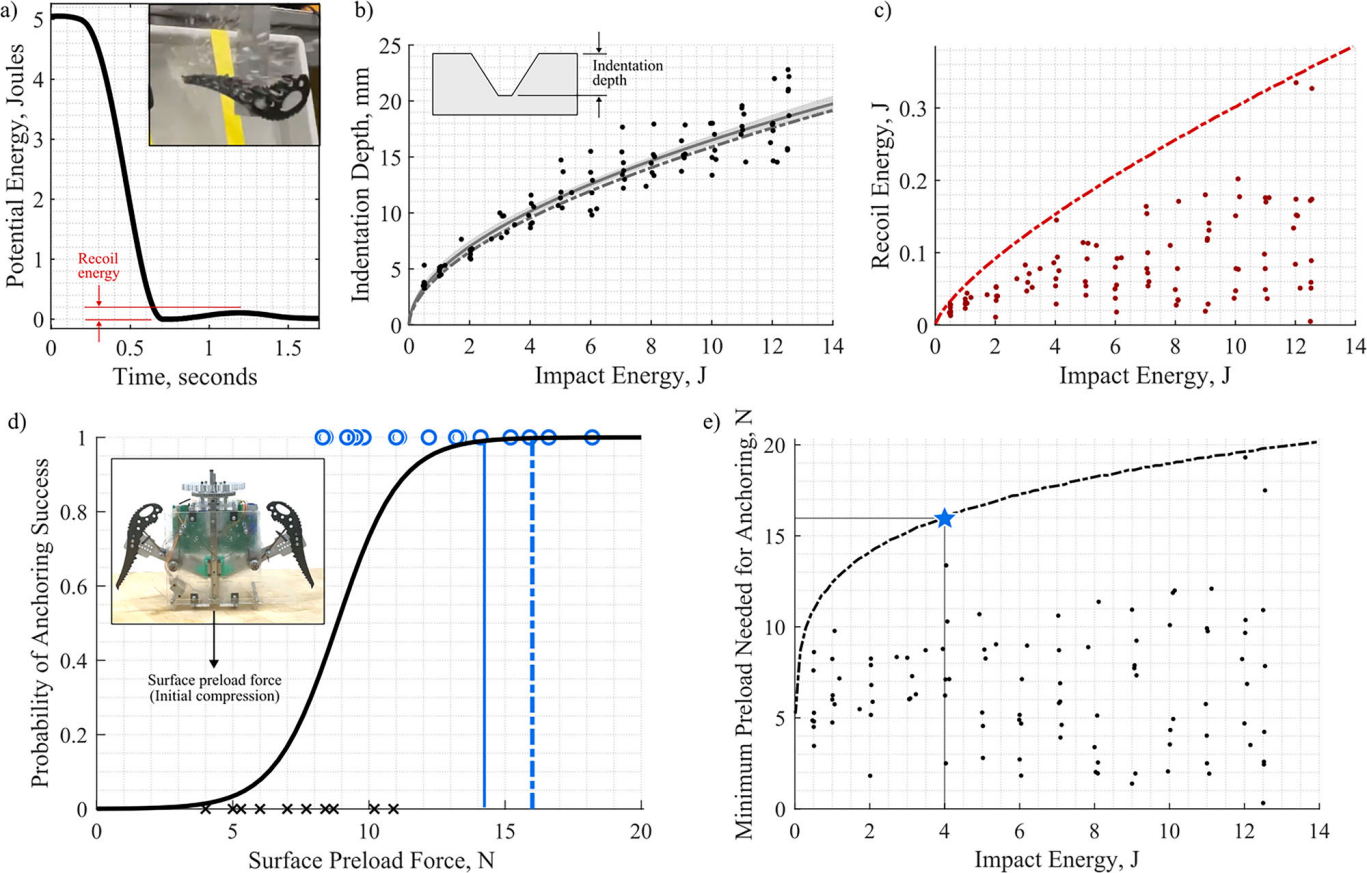
Laboratory tests predict a metric for impact grasp success based on initial compression force. **a** Time series potential energy values of a representative single-ax impact trial. **b** Indentation depth versus impact energy, showing the model prediction (dashed) and an empirical fit (solid line) with a 95% confidence interval (shaded region). **c** Recoil energy versus impact energy, showing the model prediction (dashed). **d** The gripper’s success in the impact phase across varying initial compression forces with a logistic fit and empirical 99% interval (solid vertical line). The analytical model (dashed line) predicts compression force needed for successful impact. **e** Predictions of compression preload needed for anchor success across varying impact energy.

In order to model the surface compression force needed for successful anchoring with the dual-ax gripper, we assume that each ax must stay within its indentation formed – i.e. not bounce out. By dividing recoil energy after impact, *E*_*r*_, by the depth of the indentation formed, *h*_*i*_, initial compression force needed is predicted as *F*_*i*_ = *E*_*r*_/*h*_*i*_. Depth and recoil properties are observed from single-ax pendulum measurements, as in Fig. 2a. For this single trial, the initial energy is 5.09 Joules. The ax impacts the surface, generating indentations, then bounces back with a peak potential recoil energy of 0.11 Joules. Measured indentation depths for 98 trials are shown in Fig. 2b and subsequent recoil energies in (c). An analytical model, used to predict indentation depth from input energy (b), is based on a linear low-velocity impact profile assuming a conical steel ax tip. When two parameters representing ice strength and flaw density are fit to the experimental data, they match values found in prior art^37,38^, within ± 4%, indicating that this model matches predictions accurately. The analytical model used to predict recoil energy (c) assumes Hertzian contact stiffness for a conical indenter with a spherical tip on a flat surface. While not an exact geometric match to the ax tip or ice indentation, this model appears to capture an upper bound for all measured data points. Note that most trials fall well below the predicted upper bound, however data points at impact energy 4 Joules and 12 Joules both lie close to the analytical prediction. The stochastic nature of ice fracture is well known but not well understood^39–41^. For this reason, we pursued a conservative model by assuming that all elastic deformation, at the peak force of the impact, is returned into recoil energy.

In the dual-ax gripper, each ice ax has its own dedicated spring, loaded to 4 Joules prior to impact, selected to achieve indentation depths of 1 centimeter. Both axes are identical and the motions of each ax are not mechanically coupled during the impact phase. Thus, we assume each ax acts independently on the surface and the number of axes should not effect the overall surface compression force needed prediction for the integrated system. For the selected spring energy, *E*_*r*_ = 0.16 Joules and *h*_*i*_ = 10 millimeters, such that *F*_*i*_ = 16 Newtons. This prediction is shown against 25 dual-ax trials in Fig. 2d. As expected, we observed that the impact phase of anchoring can be successful (‘o’) or unsuccessful (‘x’) depending on the amount of initial compression force between the gripper and the ice surface; higher compression forces yield a higher likelihood of success. Impact success is defined as both ax tips remaining in their indentations following impact, whereas impact failure refers to the condition in which one or both recoil out of the indentation, resulting in gripper “bouncing out.” While we observe that the gripper does succeed with as low as 8.3 Newtons of initial compression force, it also fails at as high as 10.9 Newtons. A logistic regression models the probability of impact success across a range of initial surface compression forces, and intersects the analytical model prediction of compression force needed at the 99.8% success rate. This indicates that if the gripper is provided with the initial surface compression force prescribed by the analytical model, impact success is likely. The 99% success rate taken from the empirical logistic fit is shown for comparison, at 14 Newtons. To extrapolate these results to systems with varying stored impact energy – e.g., with different spring stiffness – we plot the minimum preload needed prediction using the single ax pendulum trials in Fig. 2e, with the gripper presented in the current work indicated (star).

### Grasping phase of anchoring

**Fig. 3:**
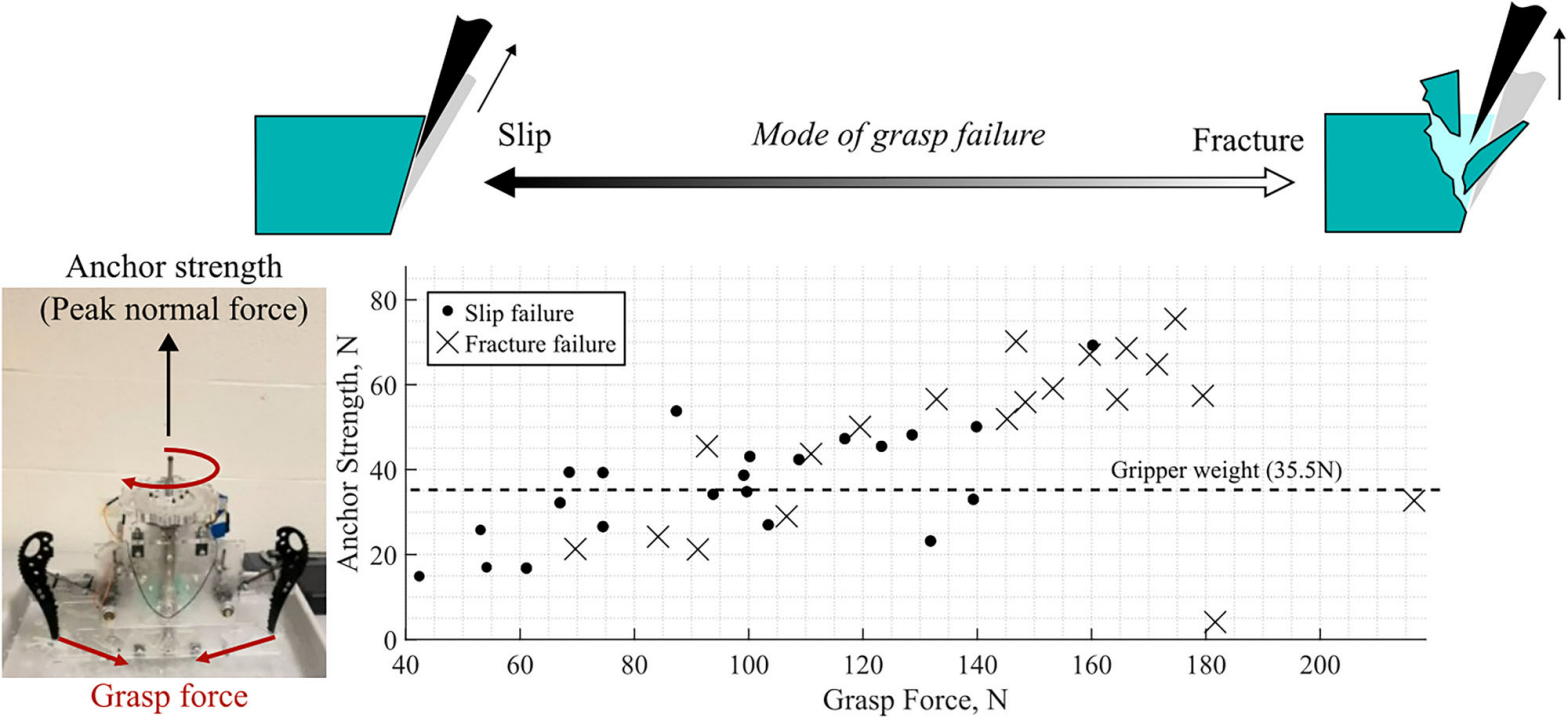
Anchoring strength performance versus grasp force. A representative test of anchoring strength shows how failure mode changes with grasp force.

The impact phase of anchoring is followed by the grasping phase, in which the gripper squeezes tangentially inward onto the indentations in order to strengthen its connection to the surface. This is accomplished with the same actuator used to store and release energy for the impact phase. During the grasping phase, the force between the ax tips is referred to as the grasp force, whereas the peak force capacity of the gripper before failure in a normal pull test is referred to as the anchor strength. The results for a series of 42 anchor strength tests with varying levels of grasp force are shown in Fig. 3. The minimum grasp force tested, 42.4 Newtons, corresponds to the squeezing force of the torsion springs alone, yielded an anchor strength of 14.9 Newtons. The maximum anchor strength observed was 75.5 N at a grasp force of 175 N; applying an internal grasp increased anchor strength by up to 5x. There is a limit to how much grasp force should be applied in order to maximize the anchor strength, however; grasp forces larger than 180 Newtons substantially decreased anchor strength. We display the observed trend of “slip failure” versus “fracture failure” of the ice. Slip failure is defined as the case in which the ice indentation ledge remains intact after losing contact with the surface. Fracture failure is defined as the case in which the gripper ax tips removes the ice ledge formed during impact. Under low grasp force conditions, slip failures become likely; fracture failures become likely under high grasp forces.

### Field testing

**Fig. 4:**
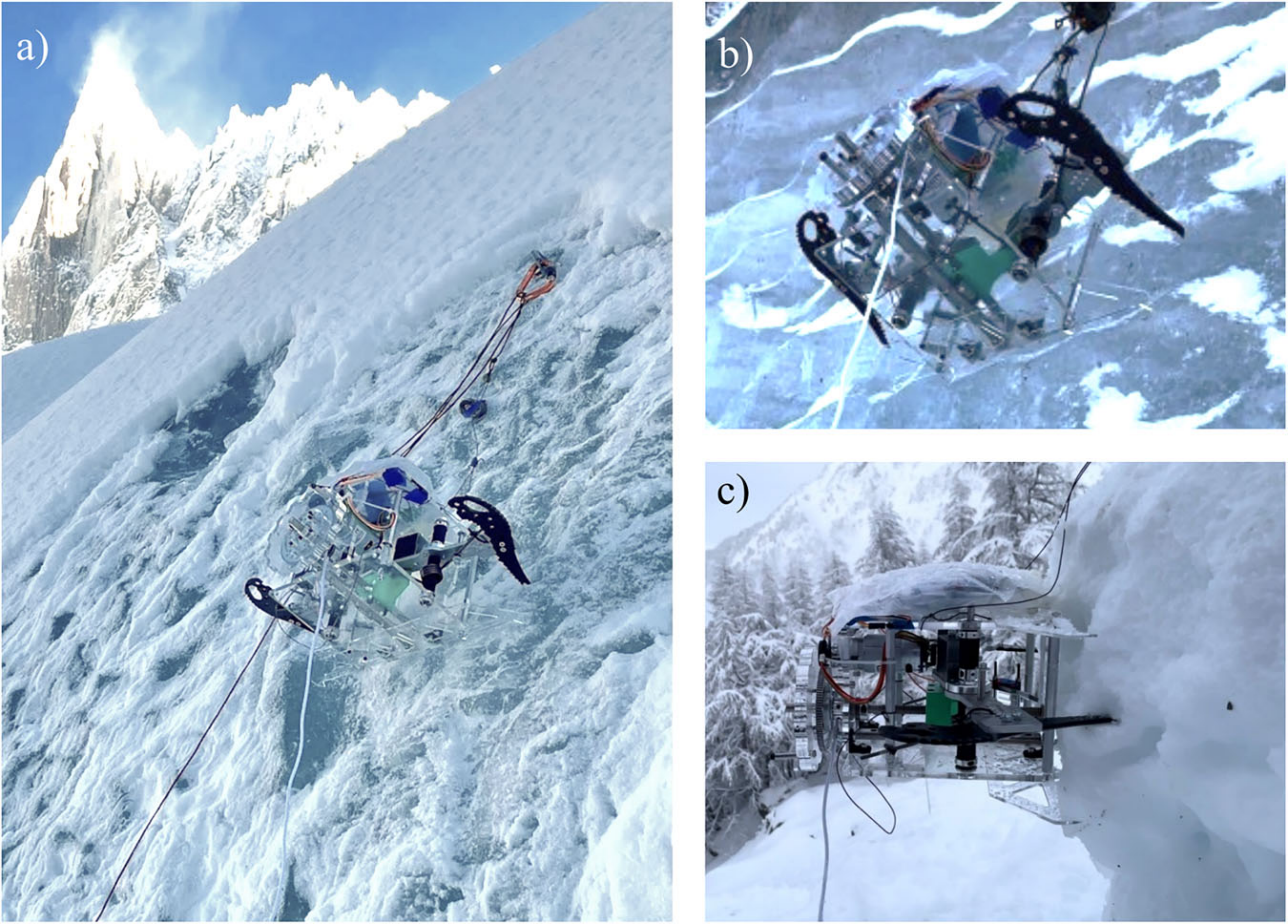
Glacier field demonstrations of the robotic impact anchoring prototype. **a** The device bearing its own weight after a successful anchoring trial on glacier ice. **b** An unsuccessful grasp phase failure on a nearly-vertical surface. **c** A successful grasp on firn.

A series of field test trials demonstrated the ability of the gripper system to form a strong anchor to natural icy surfaces with low initial compression force required. These were conducted via tethered hanging tests on glacier ice at the Mer de Glace glacier in Chamonix, France. Example field trials are shown in Fig. 4 and in Supplementary Movie 2. Overall results are reported in Table 2. Five trials were conducted on glacier ice of varying slopes, measured from horizontal. Figure 4a represents a successful grasp on a 57^∘^ slope, for which the gripper was able to bear its own weight upon the release of the tether above it. Superficial snow layers were brushed away before testing, revealing the rugose underlying ice surface. In both successful glacier trials; initial compression force was over the predicted 16 Newtons requirement and anchor strength was 70 Newtons – consistent with laboratory tests. Three trials were unsuccessful on glacier ice, as depicted in Fig. 4b. Two trials using low initial compressive preload recoiled out of the resulting indentations – again consistent with laboratory tests. One trial with an adequate compressive preload force remained in its indentations after the impact phase but failed prior to anchor strength testing; it was on a near-vertical surface and was unable to bear its own weight upon release of the tether. Note that grasp force was approximately 100 Newtons. This image also shows the smoothness and waviness of the surface, because it did not accumulate surface snow. One trial is reported on firn, or consolidated snow, shown in Fig. 4c. Not only was it is able to bear it’s own weight after grasping, but the gripper provided stronger anchoring on firn than on glacier ice likely because it was easy to penetrate.

**Table 2:** Field testing data

Ice Type	*F*_*i*_ (N)	Ice Slope (^∘^)	Impact success	Anchor Strength (N)
Glacier	6.7	90	⨯ no	–
Glacier	8.4	90	⨯ no	–
Glacier	18.8	71	✓ yes	0
Glacier	19.0	57	✓ yes	70
Glacier	18.1	57	✓ yes	70
Firn	22.9	90	✓ yes	101

Calculated initial compression force for each trial is reported along with the ice slope angle, impact success, and resulting anchoring strength (pull test to failure). All trials are actuated by the onboard motor, to a grasp force of approximately 100 Newtons.

**Fig. 5:**
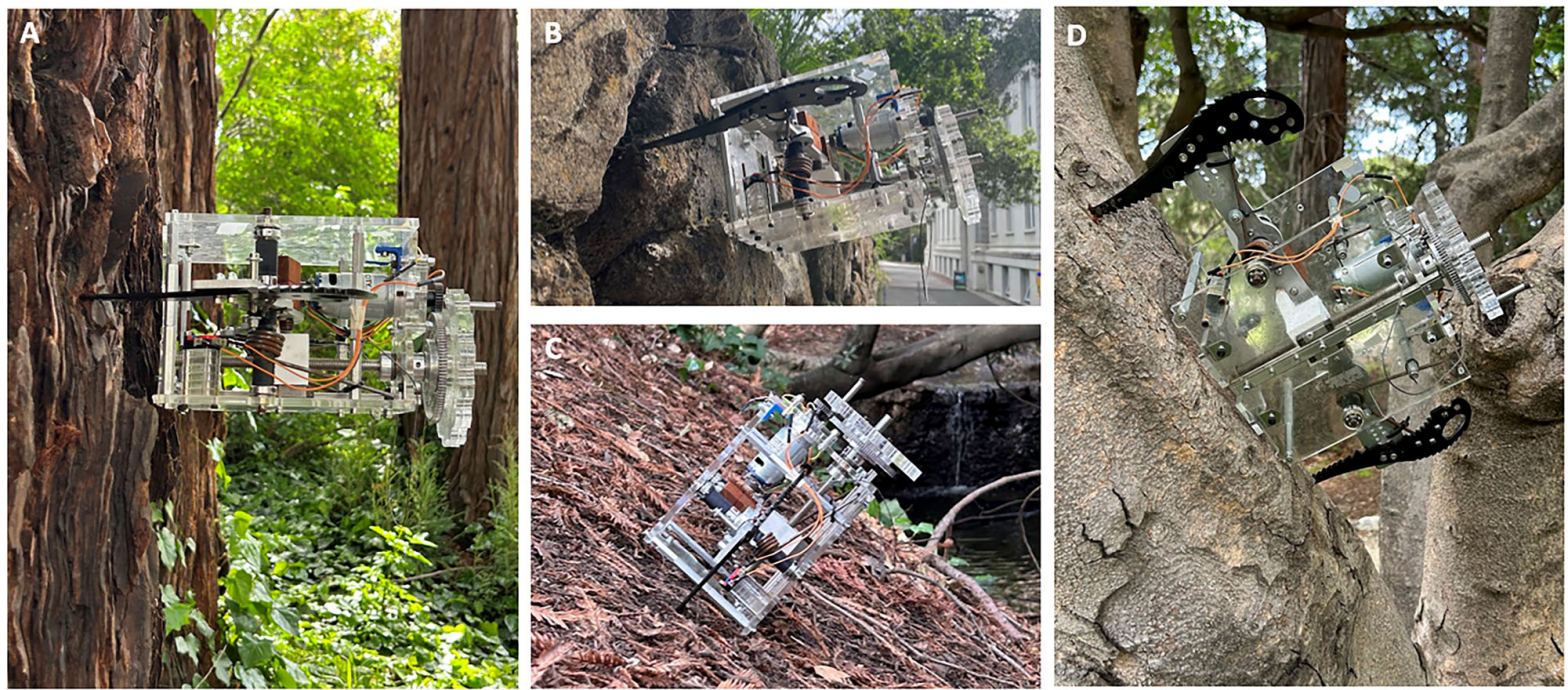
Demonstrations of the impact anchoring gripper on non-ice substrates. **A** The robotic device anchoring to a tree, and supporting its own weight. **B** The device anchoring using existing features on a rock wall, demonstrating the grasping phase on a rocky substrate. **C** A 90 N anchoring strength trial on a slope of compacted soil with detritus. **D** A 103 N anchoring strength trial to a tree.

Tests beyond freshwater ice were conducted around the University of California at Berkeley campus. Tests on various trees, compacted dirt slopes, and a rock wall are seen in Fig. 5. Across all surfaces, the gripper supported its own weight. The impact phase was used to create indentations on the trees and dirt, but only the grasp phase was used on the rocks to avoid unnecessary ax tip wear given the abundance of preexisting graspable surface asperities.

## Discussion

The integrated gripper demonstrates that symmetric impact fracture with dual ice axes can form a strong attachment to freshwater ice; a material with low strength and low fracture toughness. The advantages of the approach for mobile robotic systems that wish to anchor reversibly and repeatably include low energy expenditure per anchor formed and low initial compression force required to form an anchor, relative to other technologies. The total stored spring energy for the dual-ax gripper is 8 Joules, a two order of magnitude improvement over thermal anchoring energy expenditure. Using the conservative 16 N prediction, the initial compression force needed is at least 2x smaller than that needed for ice screws. These desired attributes were guided by potential deployment of a climbing robot at the vents of Enceladus, where gravitational acceleration is low and energy is severely limited.

We validated an energy-base fracture model and Hertzian contact model to describe the impact phase of this anchoring strategy, so it can inform future work using impact-based fracture to form attachment ice. Because we assume maximum recoil energy, our approach provides a conservative estimation of the required initial compression force to ensure that the gripper does not bounce-out of the indents in creates. We also discovered that the grasp force applied after initial impact substantially affects resultant grasp strength when pulling the anchor normally away from the surface. It is important to note that anchoring performance is sensitive to variations in ice properties related to temperature. For example, a preliminary test of the gripper on -20^∘^ C ice achieved an anchor strength of up to 163 N, 5 times the weight of the gripper itself and substantially higher than anchor strength on -14^∘^ C ice. Future work to model the mechanics of anchor failure modes on varying substrates will ultimately assist in optimizing both the design, e.g., number of axes, and control of the gripper system, e.g., grasp force. The study of factors such as the age or microstructure of the ice is also left to future work. New control approaches could also include locally perceived surface properties, such as asperity density of the ice, to provide grasp-to-grasp specific robotic predictions. From this line of reasoning, impact-based anchoring could be applied to other brittle substrates, particularly those that are otherwise incompatible or ineffective for use with microspines or gecko-inspired adhesives, e.g.,limestone or smooth sandstone.

While the current work shows the anchoring ability of a single gripper, climbing robots in the field would likely use two or more anchors at the end of different appendages to take steps while always having at least one anchor engaged. Toward this end, further characterization of the gripper performance in terms of external six-dimensional force-torque wrenches can guide appendage and robot system level design. Beyond planetary exploration at Enceladus, there are a range of other potential applications utilizing this new impact-based anchoring method. For example, a hybrid tethered-flying robot for the exploration of vertical glacier caves on Earth, such as that detailed in Ref. ^42^, could use an ice anchoring mechanism to cave walls for sampling.

## Methods

### Gripper mechanism design and components

**Fig. 6:**
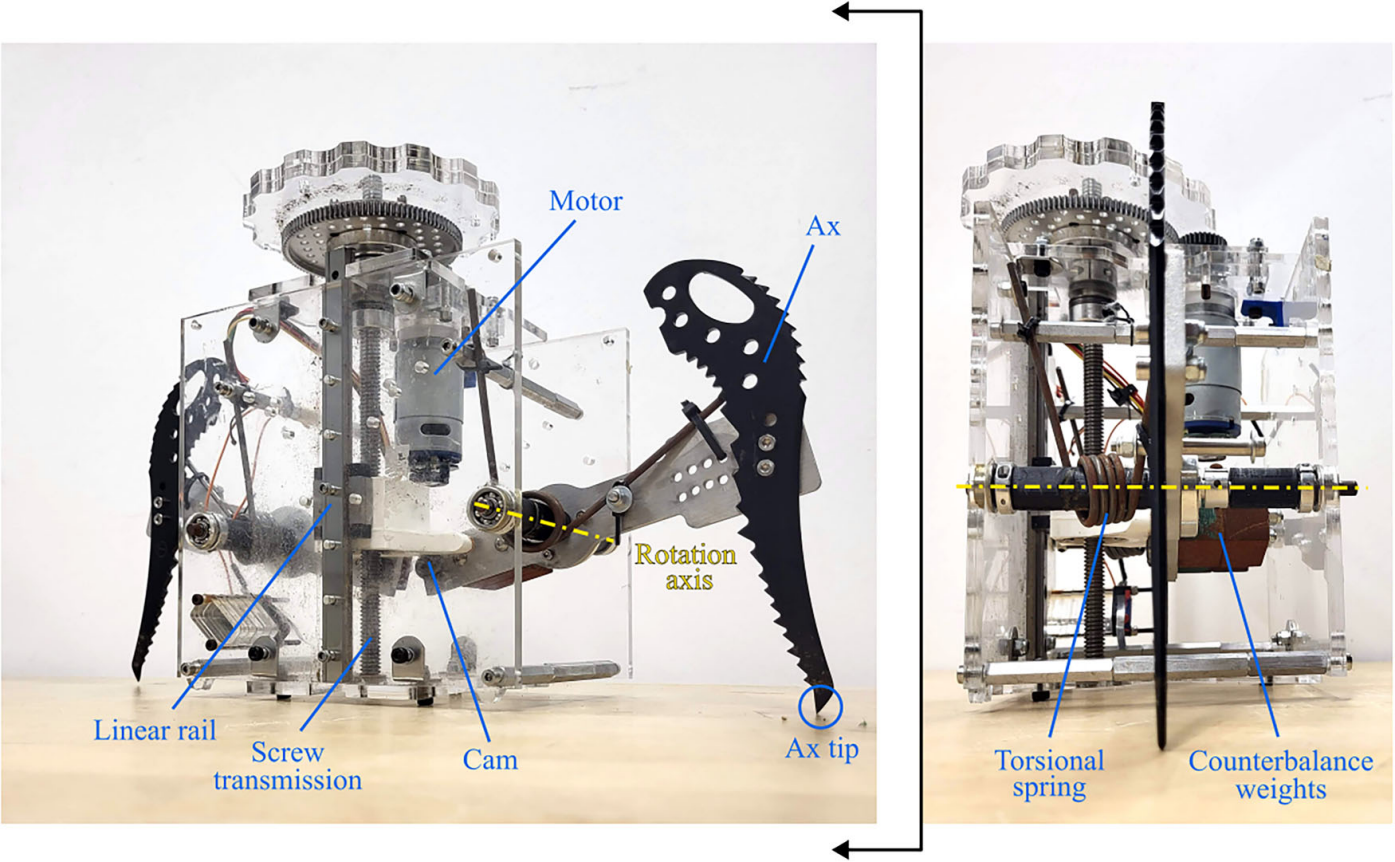
The actuation mechanisms and transmission of the ice gripper. Each of the ice axes are driven by a motorized linear screw and rail system that contacts a cam, on the same side as the counterbalance weights. The rotation axis has a torsional spring for storing potential energy.

Shown in Fig. 6, the gripper mechanism is housed in a clear acrylic frame. Each of the two waterjet 6061 T6 aluminum arms has a black-anodized steel ice ax on one end, and steel ballast weight on the other end. This counterbalance serves to place the center of mass of each arm approximately at the axis of rotation of each arm. The arms ride on one 6 mm stainless steel shaft each, mounted between two stainless steel ball bearings. The arms are spring-loaded by two torsion springs. Based on their rated stiffness and the range of motion of the mechanism, they were calculated to store 4 Joules of mechanical energy each. This amount of energy was selected based on the amount requisite to fully embed the ice ax tips roughly 1 cm below the surface. On the ballast end of each arm is a cam follower. These contact a 3D-printed PLA linear cam. The linear cam is mounted to a brass leadscrew nut internal to the cam structure, which rides on a stainless steel leadscrew with 10 mm pitch diameter, 2 mm pitch, and a single start thread. The cam is also mounted to a 10mm wide linear bearing. The leadscrew is constrained by two oil-embedded bronze bushings, one at the top of the cam’s stroke and one at the bottom of the cam’s stroke. To constrain the leadscrew axially, two thrust bearings are clamped to the top plate of the acrylic housing with steel collars mounted to the leadscrew, bearing the thrust load seen by the linear motion system. A geartrain connects the leadscrew to a 12V brushed DC motor with 7 kgf-cm stall torque and 1621 rpm no-load speed. The custom geartrain is a 3.6:1 reduction on the motor, which has a 5:1 planetary gearbox.

The two ax arms are symmetric and identical. The single linear cam engages with both arms through their own dedicated rollers. Thus, the axes move symmetrically, both driven by the same input motor. This single motor is able to actuate every phase of the gripper. During the arming phase, as depicted in this image, the cam lowers and raises both axes. Upon over-centering, the rollers slide outside the linear cam element, and the axes move freely to release stored spring energy. The cam can then be driven upwards until it engages again with the same rollers to push the ax tips closer together and applying a grasp force to the surface. When the gripper should release the grasp, reversing the actuator back down makes it easy to remove the gripper from the surface. In order to perform another arming phase, the cam then needs to over-center the rollers again by driving fully upward. The impact and grasp cycle can then be repeated.

### Predicting indentation depth and recoil for a pointed indenter impacting freshwater ice

**Fig. 7:**
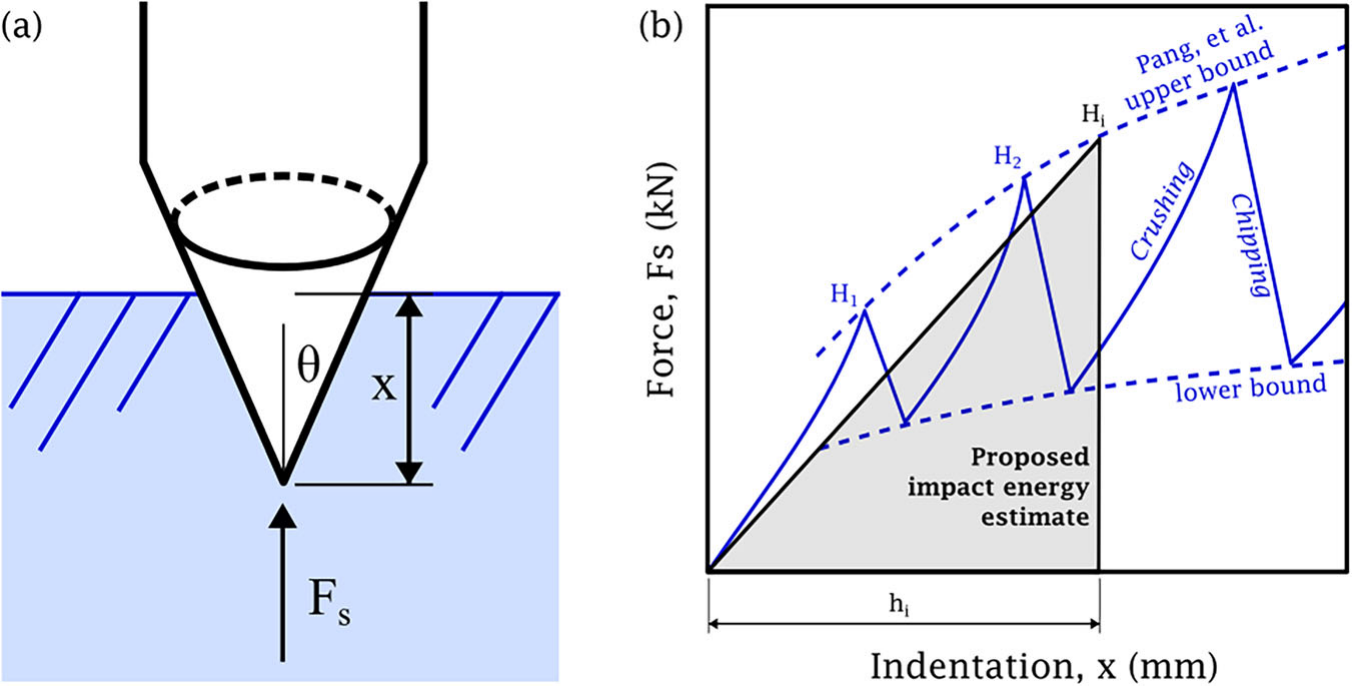
Model illustration for the peak force versus indentation depth of percussive drilling for brittle rocks. **a** The conical indenter as defined in Pang et al.^48^. **b** The crushing and chipping zones as introduced in Ref. ^48^, adapted to include an overlay of our proposed simplified method for estimating impact energy as a function of indentation depth -- the area of a triangle intersection with the upper bound of chipping.

The impact and fracture of ice is a complex interaction that depends on the ice temperature, strain rate, confinement, size, shape, and ice microstructure, among other factors^43–45^. Butin et al. studied the timeseries effects and damping of a rigid indenter impacting freshwater ice across several trials, and found that the force increases approximately linearly with indentation depth^46^. Numerical models for low-velocity rigid indenters impacting ice were presented by Yue et al.^47^ and Reitter et al.^40^ but do not match the indenter shape relevant to an ice ax. There remains a need to identify useful analytical models, in contrast to numerical models, relevant to pointed ax impact on freshwater ice. The study of impact and dynamic indentation of a pointed indenter on brittle substrates has been studied in the context of low-velocity rock penetration. Pang et al.^48^ developed a model for the peak force versus indentation depth of percussive drilling for brittle rocks as shown in Fig. 7(a). As glacier ice is considered a type of monomineralic rock, we aim to adapt this model for rock penetration to predict ax indentation on freshwater ice, using the parameters listed in Table 3. Note that the effect of fundamental mechanical differences between ice and rock in robotic impact design is left to future work. Pang et al. provide a method for modeling either wedge or cone indenters with either sharp or truncated (blunt) tips; we select the conical indenter with a sharp tip in this work because of the ax tip is roughly a tetrahedron point contact, and the ax was sharpened prior to testing. However, the ax tip does not adhere perfectly to either the conical or wedge shape and the ideal indenter model was not tested; examining the effect of such geometric variation is left to future work. Note also that the velocity range of the axes in both the pendulum impact testbed and the ice gripper itself are well within the “low-velocity” impact regime. Specifically, the estimated velocity of the gripper ax tips is 2.5 m/s based on video data and the pendulum testbed provides even slower motions. This is compared to the 10 m/s upper threshold for “low-velocity” impact^49^. Our model extends a purely force-indentation depth model to encompass impact energy required for a given depth, and introduces a model for recoil energy immediately following impact.

**Table 3:** Analytical model parameters for ice and steel, and fitted values for ice

Analytical Model Parameter Name	Analytical Model Values, Literature or Measured	Model(s) that use this parameter
**Ice Parameters**		
Reference strength of -14 deg C ice	5.5 MPa^37^ (literature)	Depth and recoil
Test specimen area of -14 deg C ice	3.14 cm^2^^37^ (literature)	Depth and recoil
Modulus of -14 deg C ice	9.33 GPa^57^ (literature)	Recoil only
Poisson’s ratio of ice	0.33^57^ (literature)	Recoil only
Weibull Parameter of ice	1.71^38^ (literature)	Depth and recoil
**Ax Parameters**		
Ax nose half-angle	10 degrees (measured by image)	Depth and recoil
Ax tip radius	0.25 mm (measured by calipers)	Recoil only
Approx. modulus of steel	200 GPa (literature)	Recoil only
Poisson’s ratio of steel	0.30 (literature)	Recoil only
**Fitted Depth Model Parameters**		
**-14 deg C Ice Fit**	**Fitted Depth Model Values**	**Percentage Error Compared to Selected Literature Value**
Fitted strength of -14 deg C ice	5.28 MPa	-4%
Fitted Weibull parameter of -14 deg C ice	1.78	+4 %

The impact energy and depth model utilizes a Weibull or flaw density parameter to characterize the effective strength of the material given the contact area. The ratio of the failure strengths of two different volumes of a brittle material is proportional to the ratio of the volumes raised to the power of a constant *m*, referred to as the Weibull parameter or flaw density parameter. Specifically, Weibull^50^ and Reichmuth^51^ found that

1 $$\frac{{\sigma }_{f1}}{{\sigma }_{f2}}={\left(\frac{{V}_{2}}{{V}_{1}}\right)}^{\frac{1}{m}}={\left(\frac{{A}_{2}}{{A}_{1}}\right)}^{\frac{1}{m}},$$

where *σ*_*f*1_ and *σ*_*f*2_ are the failure strengths of the material at volumes *V*_1_ and *V*_2_, respectively. *A*_1_ and *A*_2_ are the respective specimen effective areas. We assume that the force-indentation profile during impact is approximately linear, within the first crushing phase in Fig. 7b. The peak force *F*_1_ and maximum indentation depth *h*_1_ define the linear force versus indentation depth profile. The peak force is given by

2 $${F}_{1}={A}_{1}{S}_{1},$$

where *S*_1_ is the failure strength for the contact area *A*_1_. Assuming a conical indenter, the contact area *A*_1_ is

3 $${A}_{1}=\frac{\pi \tan \theta }{\cos \theta }{h}_{1}^{2}=C{h}_{1}^{2},$$

where *θ* is the half-angle of the conical indenter and *C* is a constant defined for use in our analysis. Furthermore, the failure strength *S*_1_ for this contact area is given by

4 $${S}_{1}={S}_{o}{\left(\frac{{A}_{o}}{{A}_{1}}\right)}^{\frac{1}{m}},$$

where *S*_*o*_ and *A*_*o*_ are, respectively, the reference strength and reference area, for a test specimen of ice. The linear force profile *F* during first chip formation can be written with respect to indentation depth *x* as

5 $$F=\frac{{F}_{1}}{{h}_{1}}x,\,\forall x\in [0,{h}_{1}].$$

and can be integrated with respect to *x* from 0 to maximum indentation depth *h*_1_ to yield the impact energy

6 $${E}_{i}={\int }_{0}^{{h}_{1}}F\,dx=\frac{1}{2}C{S}_{o}{\left(\frac{{A}_{o}}{C{h}_{1}^{2}}\right)}^{\frac{1}{m}}{h}_{1}^{3}.$$

This equation can then be written as an expression for indentation depth *h*_*i*_ as a function of the impact energy *E*_*i*_ as follows:

7 $${h}_{i}={2}^{\frac{m}{3m-2}}{\left[\frac{{E}_{i}}{{S}_{o}{C}^{\frac{m-1}{m}}{A}_{o}^{\frac{1}{m}}}\right]}^{\frac{m}{3m-2}}.$$

Note that this method approximates the nonmonotonic nature of the sequential crushing and chipping phases seen in real impact as a simple single crushing phase bounded by the upper bound of chipping, as shown in Fig. 7b.

The coefficient of restitution following impact of ice spheres has been studied in the context of the formation of Saturn’s rings^52–55^. These works shed light on the low-velocity blunt impact and coefficient of restitution of ice-on-ice interactions. In this work, we now study the relation between impact energy and recoil energy of a pointed steel indenter impacting freshwater ice. For prediction of ax recoil energy, this analysis is based on both the peak force model presented in Ref. ^48^ and the Hertzian contact stiffness of the substrate. Sharp tips, like that of the ax, may be modeled as small spheres at a miniature scale. After indenting the surface, we visually observe that ice has been removed from the surface around the point of contact, such that we assume contact rebound is dominated by the elastic deformation of the tip against a small, locally-flat surface geometry. The Hertzian contact stiffness *k* can be expressed as^56^

8 $$k=\frac{dF}{dh}\approx {\left(\frac{{E}_{* }^{2}}{RF}\right)}^{1/3},$$

for effective modulus ${E}_{* }$

9 $$\frac{1}{{E}_{* }}=\frac{1-{\nu }_{1}^{2}}{{E}_{1}}+\frac{1-{\nu }_{2}^{2}}{{E}_{2}},$$

where *E*_1_ and *E*_2_ are the Young’s moduli of each material, and *v*_1_ and *v*_2_ are the Poisson’s ratios of each material. Effective radius *R* is calculated by

10 $$\frac{1}{R}=\frac{1}{{r}_{1}}+\frac{1}{{r}_{2}}.$$

The ax tip radius is represented by *r*_1_, and the surface curvature is represented by *r*_2_. In this case, we assume an initially flat surface undergoing impact, therefore *r*_2_ is infinite. By numerically solving the differential equation for force versus displacement in Equation (8), we can produce the “unloading curve" for force and displacement and integrate with respect to indentation depth to obtain the recoil energy, *E*_*r*_. In our case, this is done numerically in MATLAB using the *ode45* and *cumtrapz* functions.

### Laboratory impact phase experiments

**Fig. 8:**
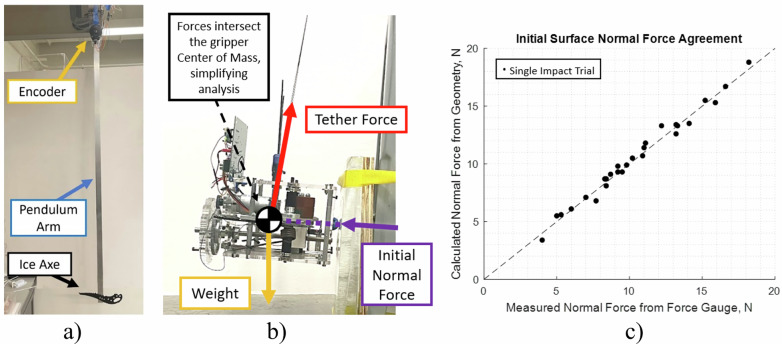
Pre-impact contact design allows for accurate initial compression force prediction based on tether geometry and gripper orientation. Designing the contact geometry of the gripper such that the forces of the tether and surface intersect at the gripper center of mass allowed for accurate computation and prediction of the initial compression force when compared with a pull test by force gauge. **a** Single-ax impact experiments are conducted using a pendulum. **b** The contact geometry of the gripper is designed such that the forces of the tether and surface intersect at the gripper center of mass. **c** This allowed for accurate prediction of the initial compression force when compared with a pull test by force gauge.

Figure 8a shows the experimental setup used for single-ax experiments, which consists of a pendulum-mounted ice ax. Sheets of ice sit at the bottom or minimum energy state of the ax, such that the impact energy depends on the initial height, or potential energy, of the pendulum. Potential energy varies in 1 Joule increments from 1 to 12 Joule, with additional trials at 0.5 and 12.5 Joules, and 7 trials at each level of impact energy. Because the pendulum is lifted manually, a live readout of the current energy helps achieve each Joule level approximately, and results in 7 trials at each impact energy level when rounded to the nearest Joule. A 10,000 count-per-revolution encoder situated at the pendulum joint is combined with manual measurements of the pendulum mass and center of mass location to enable precise measurement of the impact energy. The indentation depth and recoil energy are the dependent variables in these experiments. The indentation depth was measured with the depth rod on a pair of digital calipers relative to the surrounding surface normal. The recoil energy was taken to be the first peak in the potential energy following impact according to encoder counts.

The laboratory impact phase experiments were conducted by hanging the gripper from a tethered cable mounted to an elevated point approximately 2 meters above its contact point with a vertical block of ice, as shown in Fig. 8b. The block of ice is held rigid via ratchet straps to a heavy weighted stool. The contact geometry is a pair of half-spheres placed along the axis of the center of mass normal to the surface of ice. As a result, a static force balance is used to estimate initial surface compression force as a function of gripper weight, tether angle, and surface angle. This relation is

11 $${N}_{i}=W{\left(\sin {\alpha }_{s}+\frac{\cos {\alpha }_{s}\cos {\beta }_{t}}{\sin {\beta }_{t}}\right)}^{-1},$$

where *N*_*i*_ is the initial compression force against the surface, *W* is the gripper weight, and *α*_*s*_ and *β*_*t*_ are the surface and tether angles from the gravity vector, respectively. The comparison of this result to the initial compression force measured by a force gauge pulling on a tether behind the gripper, in Fig. 8c, demonstrating close agreement between the two methods.

The ice blocks were frozen in a commercial chest freezer at -14 ^∘^C in 18^*″*^ x 12^*″*^ x 3^*″*^ or 4^*″*^ deep aluminum pans. Ratchet straps secured each cake pan to a rigid mounting structure, which was braced against a metal desk fixed to the lab wall. Overtightening the ratchet straps introduced additional stress in the ice, that could erroneously increase recoil energy results during initial tests. Ratchet straps were subsequently tightened only to the degree necessary to constrain the aluminum pan and ice block for the loads experienced in this study. The freezing process introduced air pockets trapped in the center of the ice sheet, making indentation depths greater in this region. Thus, we collected impact data from only the more air-free regions, i.e., around the edges of the pan and at least 3 cm from the pan’s lip to reduce edge effects.

### Laboratory grasping phase experiments

The normal anchor strength experiments were conducted by placing the gripper flat on a horizontal block of ice frozen at -14 ^∘^C in an aluminum cake pan. The gripper is activated while under the normal force of its own weight, which was 33.5 Newtons for these trials. The grasp force for each trial is computed based on the mechanism characteristics and the “input force” at the handle measured with a force gauge. This corresponds to an input torque causing the gripper ax tips to grasp together. This relation depends on the chain of mechanical elements within the gripper. The torque on the leadscrew, *T*_*in*_, can be written with respect to the handle input force *F*_*in*_ by

12 $${T}_{in}={F}_{in}{r}_{in},$$

where *r*_*in*_ is the radius of the input crank from the center axis of the leadscrew. The thrust load of the leadscrew, *F*_*thrust*_ can then be found along with *β*, the lead angle, and *η*, the thread efficiency, by the following:

13 $$\beta =lead/{d}_{pitch}$$

14 $$\eta =(\cos \alpha -\mu \beta \pi )/(\cos (\alpha )+\pi \mu \beta )$$

15 $${F}_{thrust}=\frac{{T}_{in}}{\frac{lead}{2\pi \eta }+\frac{{d}_{bearing}}{2{\mu }_{bearing}}}.$$

The parameter names of each variable and their respective values and units can be found in Table 4. The grasp force *F*_*grasp*_ can be calculated by

16 $${F}_{grasp}=\frac{{F}_{thrust}{r}_{follower}}{2{r}_{tip}}.$$

**Table 4:** Mechanism parameters used for the calculation of grasp force from handle input force

Parameter Name	Parameter Symbol	Value
Handle Input Radius	*r*_*in*_	41 mm
Leadscrew Lead	*lead*	2 mm
Leadscrew Pitch Diameter	*d*_*pitch*_	10 mm
Leadscrew Coefficient of Friction	*μ*	0.3
Thrust Bearing Diameter	*d*_*bearing*_	12 mm
Thrust Bearing Coefficient of Friction	*μ*_*bearing*_	0.1
Cam Follower Moment Arm	*r*_*follower*_	92.8 mm
Ax Tip Moment Arm	*r*_*tip*_	131.7 mm

### Field impact and grasping experiments

To conduct the glacier field experiments, the gripper was hung from a tether fixed to an icy surface via a pre-deployed ice screw. The tether and surface angles were estimated from photos of each test, and used to estimate the initial surface compression force experienced by the gripper according to Equation (11). The initial surface compression force is highly dependent on tether angle, and remains relatively robust to small changes in surface angle on the order of 10^∘^ from vertical. As a result, an ice screw tether anchor point close to the gripper can result in a higher initial surface compression force for the same approximate surface angle (due to the higher tether angle relative to vertical), compared to an ice screw tether anchor point far from the gripper, resulting in a tether angle closer to vertical. Following impact, the grasp force achieved was based on motor stall at 90% duty cycle and a 7 Volt nominal voltage.

To conduct the tree trunk field experiments, the gripper was placed by hand against a tree trunk, and activated by turning the manual crank. The grasp force was then applied manually through the crank, and the gripper was shown to support its own weight on the surface. The force gauge was then used to determine the normal strength to failure. The dirt slope experiment was performed in the same fashion. The rock anchoring trial consisted of a grasping phase trial only, and was performed by manually placing the ax tips on preexisting features in the rock wall. The grasp force was then applied through the manual crank, and the gripper was observed to support its own weight on the rock.

## Supplementary information

Supplementary Information

Supplementary Video 1

Supplementary Video 2

## Data Availability

Data is provided within the manuscript or supplementary information files.
