# Supplementary material for: Fracture-based grasping: dynamic impact enables predictable robotic anchoring to freshwater ice
Source: Npj Robot. 2026 Mar 27;4(1):22. doi: 10.1038/s44182-026-00085-0 (PMC13031120; doi:10.1038/s44182-026-00085-0)
Supplement: Supplementary file 1 — Supplementary Information [file 44182_2026_85_MOESM1_ESM.pdf]

Supplementary Information

Fracture-based grasping: Dynamic impact enables predictable robotic anchoring to freshwater ice

Movies

Supplementary Movie 1: GripperOperations.mp4

This video resource presents the operation modes of the ice ax gripper. From 00:00 to 00:03, the gripper demonstrates the arming phase on a flat sheet of ice. From 00:04 to 00:07, the impact phase occurs at 0.5x speed. This arming and impact phase trial is repeated starting at 00:08 for a vertical ice surface in the laboratory at 1x speed. The reset phase is demonstrated starting at 00:15.

Supplementary Movie 2: GlacierTrial.mp4

This video is a real-time (1x speed) playback of one complete gripping trial conducted on a glacier slope. The gripper initially hangs from a tether against the ice slope. After the impact and gripping phases, the ability of the gripper to support its own weight is demonstrated starting at 01:04 by the release of the tether from which it was hanging at the beginning of the trial.

Tables

Supplementary Tables 1, 2, and 3 are provided on the following pages.

Supplementary Table 1

Fracture-based grasping: Dynamic impact enables predictable robotic anchoring to freshwater ice
Data Cooresponding to Figures 2b, c, and e

Impact Energy (J)	Depth (mm)	Recoil Energy (J)	Minimum Preload Needed (N)
0.47	3.50	0.02	4.86
0.50	3.80	0.03	7.61
0.50	5.33	0.02	4.50
0.51	3.25	0.03	8.62
0.51	3.60	0.02	5.28
0.51	3.54	0.02	4.80
0.51	3.76	0.01	3.46
1.00	4.37	0.04	8.24
1.00	4.85	0.02	4.74
1.00	4.83	0.03	6.00
1.01	5.13	0.03	6.24
1.06	4.50	0.04	9.78
1.06	5.22	0.03	5.75
1.19	5.32	0.04	7.17
1.73	7.65	0.04	5.48
2.01	6.00	0.01	1.82
2.03	6.59	0.03	5.16
2.03	6.30	0.05	8.25
2.03	6.71	0.05	7.90
2.04	5.88	0.04	6.80
2.08	6.80	0.04	5.88
2.72	7.67	0.06	8.34
3.00	10.00	0.08	8.30
3.05	7.80	0.05	6.03
3.08	9.73	0.06	6.06
3.13	9.74	0.07	7.29
3.23	8.26	0.05	6.30
3.49	8.95	0.08	8.72
3.95	9.79	0.09	8.78
4.01	8.67	0.05	6.23
4.02	9.00	0.06	7.11
4.03	11.59	0.03	2.50
4.04	10.84	0.15	13.38
4.07	9.13	0.09	10.30
4.12	10.54	0.07	7.13
4.93	10.66	0.11	10.69
5.00	11.34	0.06	5.29
5.02	14.73	0.04	2.80
5.02	11.86	0.05	4.55
5.06	10.45	0.09	8.75

5.09	13.68	0.11	8.26
5.37	11.82	0.10	9.04
5.99	10.20	0.05	4.89
6.00	13.63	0.04	2.72
6.00	15.50	0.08	5.16
6.04	9.81	0.02	1.83
6.04	11.80	0.06	4.69
6.06	12.91	0.09	7.13
6.20	10.37	0.09	8.97
7.03	15.45	0.16	10.62
7.05	17.67	0.15	8.72
7.05	13.42	0.08	5.81
7.08	12.21	0.07	5.90
7.08	14.49	0.10	6.90
7.09	13.78	0.05	3.92
7.12	13.00	0.06	4.62
7.83	12.38	0.11	8.89
8.00	14.45	0.05	3.39
8.03	13.60	0.03	2.02
8.07	15.22	0.08	5.13
8.09	17.94	0.04	1.95
8.09	13.34	0.03	2.55
8.11	15.03	0.17	11.38
9.00	13.72	0.02	1.39
9.00	16.45	0.18	10.94
9.06	15.14	0.12	7.73
9.07	15.16	0.12	7.89
9.09	14.92	0.03	1.94
9.12	17.86	0.13	7.34
9.12	15.26	0.14	9.24
9.96	18.00	0.04	2.06
10.01	13.37	0.05	3.54
10.01	14.96	0.15	10.09
10.02	18.01	0.08	4.33
10.09	17.02	0.20	11.87
10.10	15.60	0.08	4.94
10.14	14.78	0.18	12.00
10.97	17.02	0.10	5.76
10.99	19.37	0.08	4.02
11.00	17.74	0.18	9.92
11.00	19.58	0.05	2.50
11.02	17.42	0.17	9.76
11.06	18.83	0.04	1.93
11.12	14.55	0.18	12.10

11.94	16.28	0.13	8.23
12.00	17.90	0.08	4.69
12.02	18.00	0.17	9.67
12.02	17.35	0.34	19.31
12.03	14.65	0.15	10.38
12.07	22.00	0.15	6.86
12.16	14.54	0.05	3.51
12.50	15.62	0.01	0.32
12.50	15.75	0.17	10.92
12.52	20.90	0.05	2.44
12.52	22.80	0.06	2.59
12.53	21.05	0.09	4.23
12.55	22.18	0.17	7.85
12.55	18.69	0.33	17.50

Supplementary Table 2

Fracture-based grasping: Dynamic impact enables predictable robotic anchoring to freshwater ice
Data Cooresponding to Figures 2d and 8

Surface Preload Force, from Force Gauge (N)	Anchor success	Surface preload calculated from Geometry (N)
4	FALSE	3.4
7.7	FALSE	6.8
18.2	TRUE	18.8
12.2	TRUE	13.3
9.8	TRUE	9.9
10.2	FALSE	10.5
8.4	TRUE	8.7
8.3	TRUE	8.7
8.4	FALSE	8.1
13.3	TRUE	13.3
13.2	TRUE	13.4
13.2	TRUE	12.6
5	FALSE	5.5
5.3	FALSE	5.6
7	FALSE	7.1
9.2	TRUE	9.3
11.1	TRUE	11.8
9.5	TRUE	9.3
9.2	TRUE	9.8
8.7	FALSE	9.1
15.9	TRUE	15.3
15.2	TRUE	15.5
11	TRUE	11.4
10.9	FALSE	10.7

Supplementary Table 3

Fracture-based grasping: Dynamic impact enables predictable robotic anchoring to freshwater ice
Data Cooresponding to Figure 3

**Actuation force on input handle (N)**	**Anchor strength (N)**	**Failure type (F=Fracture, S=Slip)**
6	14.9	S
8	25.8	S
8.2	17	S
9.5	16.8	S
10.6	32.2	S
10.9	39.4	S
11.1	21.3	F
12	26.6	S
12	39.3	S
13.8	24.2	F
14.4	53.8	S
15.1	21.2	F
15.4	45.5	F
15.6	34.2	S
16.6	38.7	S
16.7	34.8	S
16.8	43.1	S
17.4	27	S
18	29	F
18.4	42.4	S
18.8	43.7	F
19.9	47.3	S
20.4	50.1	F
21.1	45.5	S
22.1	48.2	S
22.7	23.2	S
22.9	56.6	F
24.1	33	S
24.2	50.1	S
25.2	51.9	F
25.5	70.2	F
25.8	55.9	F
26.7	59.1	F
27.9	67.1	F
28	69.3	S
28.8	56.5	F
29.1	68.6	F
30.1	64.8	F
30.7	75.5	F

31.6	57.4	F
32	4.1	F
38.5	32.7	F
